# Beyond the surface: untangling molecular complexity in organic residue analysis of coated archaeological ceramics

**DOI:** 10.1098/rsos.250639

**Published:** 2025-10-15

**Authors:** Jasmine Lundy, Julia Haas, Birgit Öhlinger, Léa Drieu

**Affiliations:** ^1^ Department of Archaeology, University of York, York, UK; ^2^ Department of Archaeologies, University of Innsbruck, Innsbruck, Austria; ^3^ Université Côte d'Azur, CNRS, CEPAM, Nice, France

**Keywords:** organic residue analysis, post-firing coating, archaeological ceramics, experimental

## Abstract

Mixed molecular and isotopic signals are readily encountered in organic residue analysis (ORA) of archaeological ceramics, which can impact our ability to identify the contents/use of the vessel. One reason for these mixed signals that is rarely considered is the mixture of organic products used to coat vessels and the organic products that were later contained in the ceramic vessel. Here, we apply a multifaceted ORA approach to experimental cooking vessels that were coated with a range of organic products during post-firing treatment and then used to cook a variety of organic contents. Our results show that the visibility of the coating agent or cooking commodity varies depending on the type of extraction or analytical method applied. We show that it is important to use a combination of extraction methods (acidified methanol and solvent extraction) and that both molecular and isotopic data must be considered. For the first time, we have also shown that thermal transformation markers could reflect the post-firing coating as opposed to cooking. We call on ORA specialists to carefully consider the impact of post-firing coating before interpreting residues from archaeological ceramics and to implement a multifaceted ORA approach to aid their detection.

## 1. Introduction

In organic residue analysis (ORA) of archaeological ceramic containers, it is not uncommon to identify mixed chemical signatures. They can result from (i) the intentional mixing of products as part of a single recipe; (ii) the accumulation of sequential uses of the vessel for different products [1]; or (iii) a mixed signal reflecting both technological uses (coating the vessel with organics) and the organic products that were cooked in the vessel for culinary enjoyment. The latter has been addressed by a handful of studies [2–5] which investigated the use of post-firing technologies to understand how these may be identified in archaeological ceramics. These previous works have shown that organic surface treatments can leave a molecular signal in the walls of pottery, and when the organic coating is applied to hot ceramic surfaces, this molecular signal is altered compared with the raw product, leaving thermal transformation markers (i.e. mid-chain ketones). It has also been demonstrated that only partial waterproofing is achieved by post-firing coating, and in archaeological samples a mixed signal may be attributed to the mixture of the coating agent and the contents of the vessel that have been absorbed despite coating.

However, to fully understand the post-firing coating signals observed in archaeological ceramics, these first results require expansion in several ways. Firstly, these investigations focused only on a limited range of coating products (pine resin, lard and bark decoction) and contained products (olive oil and wine). However, there are a great number of other commodities that have been successfully detected in archaeological pottery by ORA, of which some (e.g. milk) have been regularly cited as potential waterproofing products [6–8]. Secondly, this issue has primarily been tackled using a molecular approach, while the identification of foodstuffs in ceramics through ORA now largely involves single-compound stable carbon isotope analyses. Finally, in previous studies acidified methanol extraction (AE) [9,10] which is now the most common extraction method in ORA, has never been applied to address this issue. AE extracts both free and bound lipids, providing deeper insight into all lipids related to the history of the pot [9]. In particular, it can more efficiently extract potential by-products of post-firing coatings applied to hot surfaces, such as diacids and hydroxy acids (as suggested in a previous study [9]), as well as additional thermal transformation markers, such as ω-(o-alkylphenyl)alkanoic acids (APAA) [10]).

In this study, we aimed to widen our understanding of the impact of different post-firing coatings on our interpretations of organic residues in archaeological ceramics by addressing the following questions: Do products other than lard and resin leave a molecular signal in ceramic walls when used as a coating agent on hot surfaces? Does AE provide additional molecular information about the surface coating? Can the comparison between conventional solvent extraction (SE) and AE provide information to distinguish between surface coating and content? Do single-compound stable carbon isotope values provide information about the surface coating or the contents of the vessels?

Here, we designed our methodological approach to build on previous research by applying a range of coating products such as milk, beef tallow and a mixture of beef tallow and pine resin to the hot surfaces of experimental pottery vessels. A variety of foodstuffs (fish, vegetables, ruminants, dairy and non-ruminant animal products) were then cooked in coated vessels and uncoated vessels for comparison. To fully assess the impact of different post-firing coatings and products, the experimental ceramic vessels were analysed using a multifaceted ORA approach, including SE and AE as extraction methods and molecular and isotopic analyses.

## 2. Material and methods

### 2.1. Post-firing coating and cooking experiments

**Figure 1.**
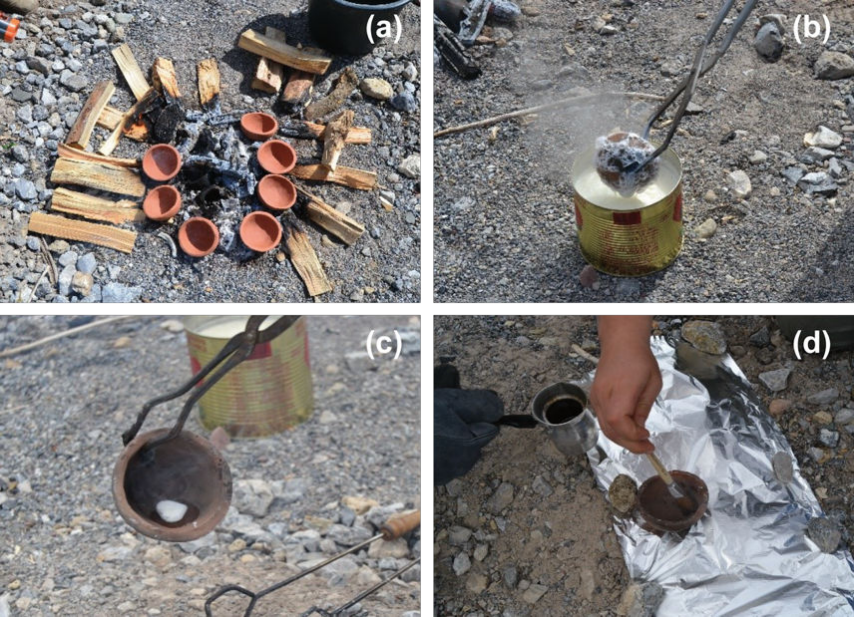
Coating experiments: (a) field fire heating the vessels; (b) dipping the vessel into the milk; (c) applying the beef tallow; and (d) applying the pine resin.

Cooking experiments were performed as part of the Monte Iato Pots experimental project funded by the Early-Stage Funding of the University of Innsbruck. The full details of these experiments are outlined in electronic supplementary material, S1. In brief, replica cooking pots were made from clay collected near the site of Monte Iato, Western Sicily (for research on the site, see https://www.uibk.ac.at/projects/monte-iato/index.html.en, with a list of publications; for ORA of archaeological samples, see M Mohr & F Notarstefano 2017, unpublished data; [11]). The clay was prepared for processing and shaped in small hemispherical plaster moulds without the addition of further additives to the raw material. The surface of the bowls was then slightly smoothed. The vessels were fired at 800°C for 24 h in an electro kiln. A number of these replica vessels were then coated with different commodities: milk; beef tallow; a mixture of pine resin and beef tallow. The products were applied on the fired pots immediately after being reheated in a field fire (figure 1a) by either submerging in the coating product or generously coating the ceramics. For the milk coating, an untreated product from a Tyrolean dairy farmer was used. After being heated (300–500°C) within the field fire, the vessels were dipped completely into the milk three times (figure 1b). The beef tallow was made up of beef fat from a Tyrolean cow, heated to 47°C and filled into 5 mL moulds to ensure that the same quantity of beef tallow was used for all coating processes. After heating the vessels to around 300–400°C, the portion of beef tallow was applied to the inner surface by panning (figure 1c), which turned dark brown (electronic supplementary material, figure S1b). The pine resin was collected in the woods of Tyrol and mixed with a bit of beef tallow to make it easier to apply to the inner surface of the hot vessels (around 300°C) with a brush (figure 1d), which turned darker but not as dark as with beef tallow (electronic supplementary material, figure S1c). Some vessels were left uncoated for comparison purposes.

**Figure 2.**
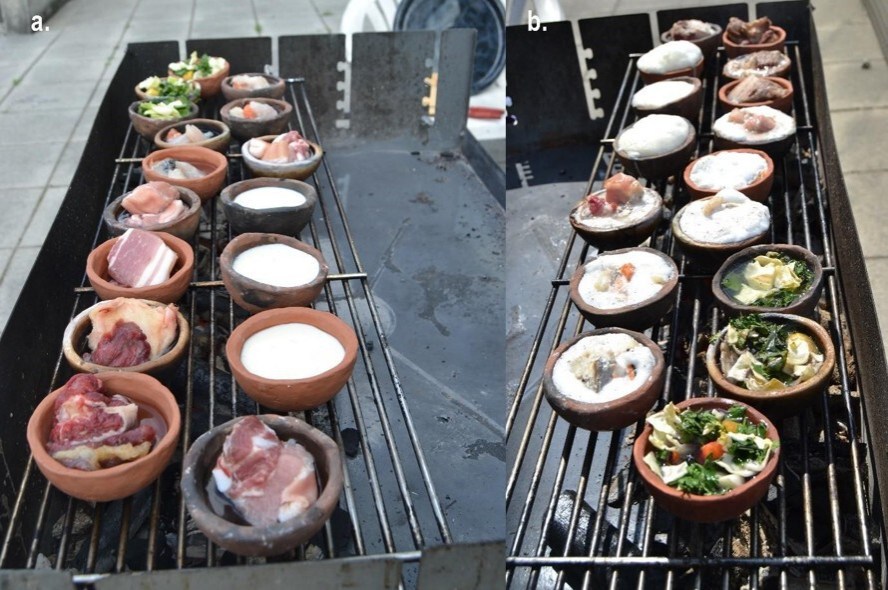
Experimental pots with different organic products (a) before and (b) during cooking.

Various products were then cooked in the coated and uncoated ceramics. For the cooking process, organic milk, pork, beef, vegetables and fish of Mediterranean origin (redfish, sea bass, coalfish, salmon and anchovy paste) were cooked over an open coal fire for 10 h (2 consecutive days for 5 h each) reaching temperatures of 80–100°C within the vessels and 120–150°C on the outer surface (figure 2).

After cooking, any leftovers were removed with a spoon and the ceramics were wrapped in tin foil. To avoid contamination, the vessels were only handled with gloves during the entire experiment and placed on or wrapped in tin foil.

### 2.2. Organic residue analysis

The experimental ceramics were analysed using well-established extraction methods and analytical techniques for archaeological ceramics. In brief, the ceramic sample was first ‘cleaned’ by removing the outer layer of the ceramic surface with a clean Dremel drill bit (approx. 1 mm deep) to avoid exogenous contamination. Although exogenous contamination from the burial environment is not a concern here and handling contamination is low, the removal of the outer surface is important to achieve the most comparable results to archaeological samples. Approximately 2 g of ceramic powder was collected by drilling into the ceramic pot. Two extraction methods were then applied: an AE method [9,12], now the most commonly used method in archaeological samples that favour both high extraction yield and direct methylation for stable carbon isotopes measurements and a SE [13], to ensure the preservation of the most-informative molecules in fatty and waxy products (triacylglycerols and wax esters). Both AE and SE extracts were characterized using gas chromatography (GC) and gas chromatography–mass spectrometry (GC-MS) techniques. To further understand the origin of these lipids, AE extracts were analysed by Gas Chromatography-Combustion-Isotope Ratio Mass Spectrometry (GC-C-IRMS) to determine the stable carbon isotope value of the major fatty acids (C_16:0_ and C_18:0_) and identify animal resources based on a comparison of the δ^13^C values with modern reference values [6,12,14,15]. The equipment and instrument parameters are as previously described [16] and are outlined in electronic supplementary material, S2. A blank ceramic sample (uncoated and unused) was extracted following the same procedures to ensure that no lipids were coming from the unfired clay [17].

## 3. Results and discussion

### 3.1. Uncoated vessels: identifying contents through organic residue analysis

For uncoated vessels used to cook or contain one type of commodity, the molecular profiles obtained are per the data published in the literature for animal adipose fats, milk and resin. These extracts were dominated by free fatty acids, where palmitic (C_16:0_) and stearic (C_18:0_) acids were most dominant. Linear and branched odd carbon-numbered fatty acids (mainly C_15:0_ and C_17:0_) were detected in the majority of samples, in particular in the pots that contained ruminant adipose fats (RAF, including tallow) and milk. Furthermore, branched odd-numbered chain fatty acids were observed in the vessel used to cook non-ruminant adipose fats (NRAF). This could be explained by the fact that these animals may have consumed dairy products, and highlights that caution needs to be taken when interpreting RAF based on the presence of branched-chain fatty acids [18]. More generally, branched fatty acids should be interpreted with caution since they are produced by microbial activity and may therefore indicate contamination and/or degradation [15,19]. A series of short-chain α,ω-dicarboxylic acids (diacids; resulting from the degradation of unsaturated fatty acids) [18] were present in several samples, ranging from carbon atom lengths C_7_ to C_14_. The sample that contained fish products was also dominated by unsaturated fatty acids (C_16:1_, C_18:1_, C_20:1_, C_22:1_, C_24:1_). After selected ion monitoring methods, isoprenoids phytanic, pristanic and 4,8,12-trimethyltridecanoic acid, typical in aquatic resources [10,20], were also detected. Probably due to its low lipid yield, the vessel that contained vegetable products produced a simple lipid profile with only minor compounds, predominantly made up of palmitic and stearic acid with some contribution of C_18:1_.

The triacylglycerol (TAG) profiles in SE extracts were also a strong marker of the type of contents, in line with the published literature [15,21], whereby the vessel used to cook milk showed a very broad profile (T_28_–T_54_), while vessels used to cook carcass fats had much narrower profiles. Within these profiles, we could distinguish the profile of ruminant carcass fats, which was moderately broad (T_46_–T_54_), centred on T_52_, and that for porcine carcass fats, which was very narrow (T_48_–T_54_) and largely dominated by T_52_ as described in previous studies [22]. The TAGs of fish are made up of a large proportion of unsaturated fatty acids, which cannot be distinguished using conventional GC-MS and no TAG profiles for fish oils and vegetables in archaeological contexts are available in the literature. Here, TAGs observed in vessels used to contain fish may be interpreted as RAF based on their short profile, centred around T_52_ and no TAGs were detected in the vessel that contained vegetables.

GC-C-IRMS analysis was used to determine the δ^13^C values of the major fatty acids (C_16:0_ and C_18:0_) of extracts from pottery without a coating agent. It must be noted that organic products used to cook in pottery were not selected with dietary or environmental conditions in mind. Therefore, the δ^13^C values obtained here cannot be directly used as authentic references to compare with δ^13^C values from archaeological extracts. Although the samples used to cook RAF (cattle belly fat), showed a Δ^13^C in the typical range of ruminant carcass fats (less than −1.0 and greater than −3.3‰ [6,18]), the δ^13^C_16:0_ and δ^13^C_18:0_ (−23.77 and −24.61‰, respectively), suggest that the animals were fed on a mixed, unquantifiable, C_3_ and C_4_ diet, possibly including silage [23]. The pottery sample containing milk yielded δ^13^C_16:0_ and δ^13^C_18:0_ similar to previously reported values of modern dairy (−22.59 and −29.69‰, respectively) and a Δ^13^C value less than −3.3‰ (−7.09‰). The isotopic results from the pottery vessel used to cook fish products are reflective of the mixture of fish cooked in the same vessel (redfish, sea bass, coalfish and salmon): δ^13^C_16:0_ = −25.77 and δ^13^C_18:0_ = −26.39‰. The vessel used to cook NRAF (porcine belly fat) showed typical δ^13^C_16:0_ and C_18:0_ values of European porcine (−25.00 and −24.09‰, respectively) and a Δ^13^C value similar to other modern non-ruminant carcass reference values greater than −1.0‰ [23,24]. The pottery vessel used to cook mixed vegetables did not yield appreciable concentrations of C_16:0_ and C_18:0_ for reliable measurements. Lipid profiles and single compound stable carbon isotope values per sample are detailed in electronic supplementary material, S3 and GC-MS files at [25].

### 3.2. Coated vessels: observing the impact of post-firing surface coatings

The molecular and isotopic signatures obtained on coated vessels, used and unused, were highly variable from one sample to another. This variability was probably due to multiple parameters that are not easy to control in the experiments, such as variable surface temperatures, non-homogeneous minerals and porosity in the ceramic paste, and biases due to filling level variations, splashing and capillarity phenomena [26,27]. In particular, the mechanisms of absorption and degradation during coating are difficult to assess, and it is possible that, during the process of the post-firing coating, high temperatures will have led to the partial degradation of lipids of the coating product. These unknown mechanisms of absorption and degradation become even more difficult to assess in archaeological ceramics. Despite this variability, it is possible to highlight some significant results.

#### 3.2.1. Quantitative effect of post-firing coating

**Figure 3.**
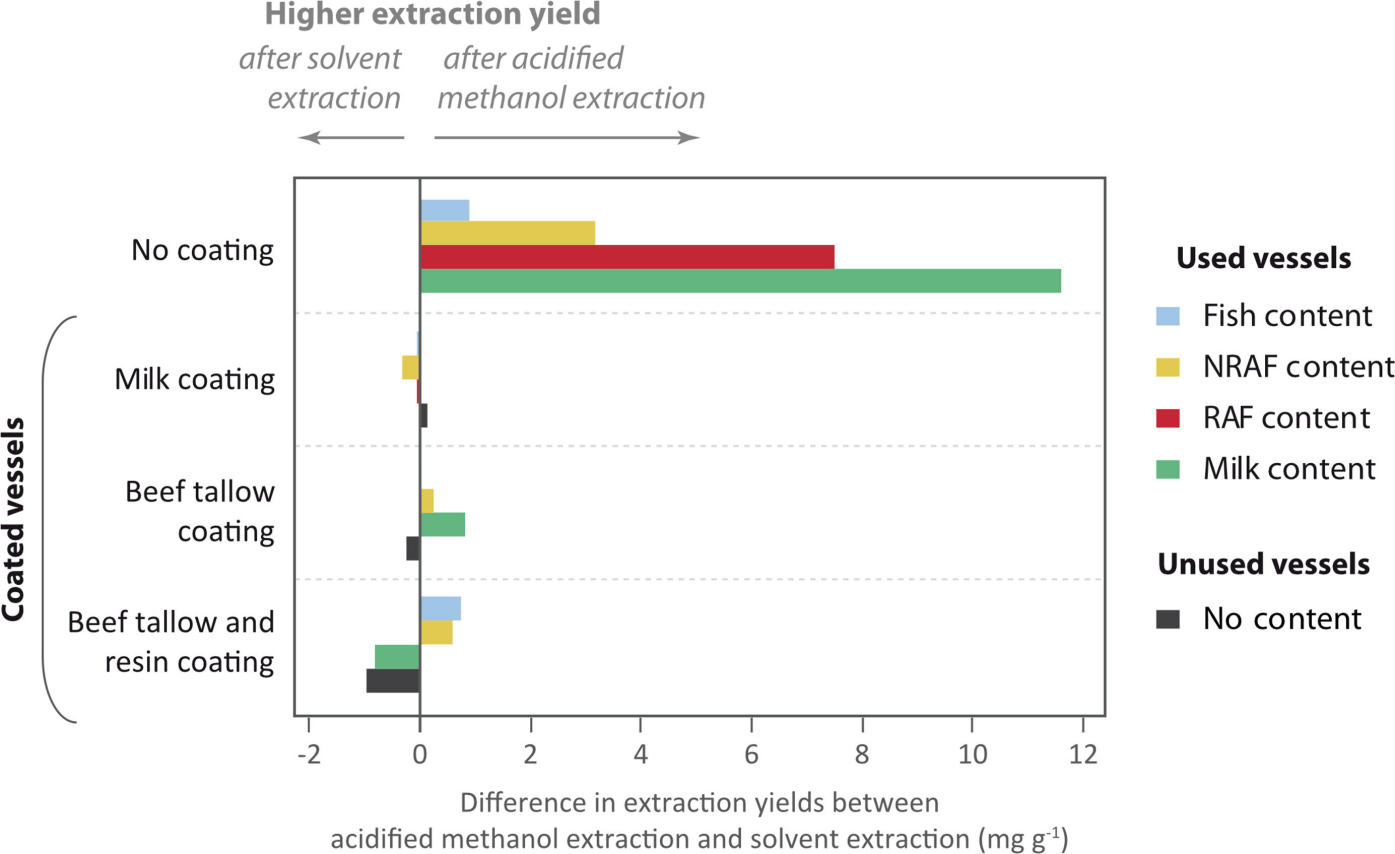
Comparison of extraction yields between acidified methanol extraction and solvent extraction.

**Figure 4.**
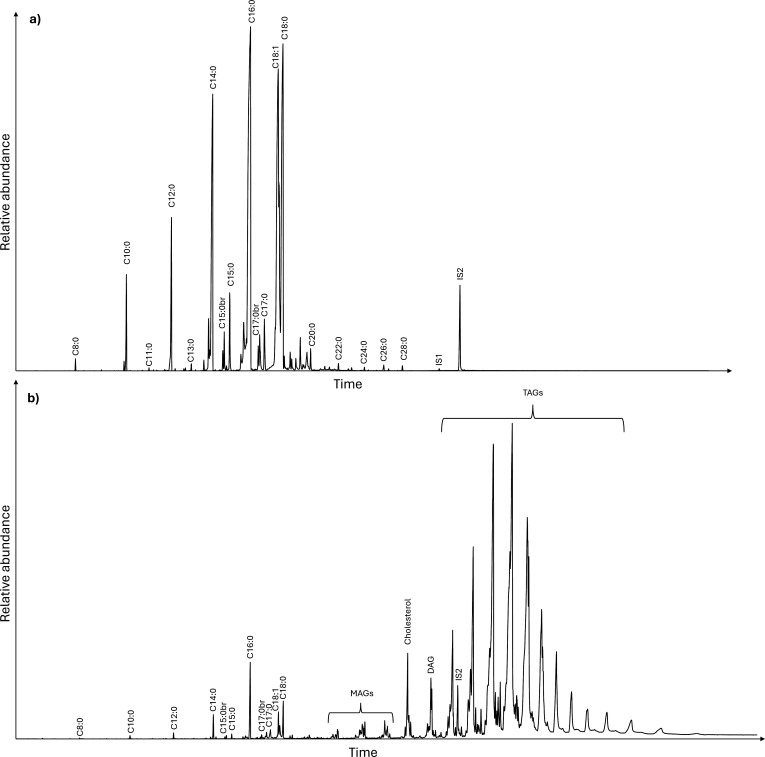
Total ion chromatogram of (a) AE of sample 9 and (b) SE of sample 9. Showing the main fatty acid components (Cn:x), Monoacylglycerols (MAGs), diacylglycerols (DAGs) and TAGs and internal standards (IS1 and IS2).

Extraction yields in coated but unused vessels (between 0.1 and 1.5 mg g^−1^ of lipids) confirmed that coating of hot surfaces with organic substances leaves molecular markers in the ceramic walls as previously shown (M Mohr & F Notarstefano 2017, unpublished data). AE significantly increased extraction yields from used uncoated containers compared with SE (figure 3), in line with previous results [9]. In contrast, this was not observed to such an extent for coated vessels (figures 3 and 4). One possible explanation is that the coating prevented the formation of chemical bonds between the pottery and the contents. This would mean that AE was not necessary to break these bonds, resulting in similar extraction yields after AE and SE. This could be investigated further by comparing the isotope values after SE and after AE to confirm whether the extracts have similar values. In addition, degradation experiments should be carried out by varying parameters (pottery fabric, soil pH, humidity, etc.) to check that taphonomic mechanisms do not alter these results. Further experimentation would also be required to verify that multiple repeated uses may not generate a similar effect.

#### 3.2.2. Qualitative effect of organic coating

##### 3.2.2.1. Identification of the coating product

After SE, the unused milk-coated vessel showed a molecular profile dominated by a broad profile of TAGs (T_32_–T_54_) typical of dairy products [26]. Apart from the loss of very short TAGs from fresh milk (T_28_ and T_30_), the TAG profile was only slightly altered by coating on hot surfaces. Free fatty acids were mainly composed of palmitic acid and short-chain fatty acids (C_8:0_–C_14:0_). Stearic and oleic acids were present in small amounts, as well as odd-chain linear and branched fatty acids (C_15:0_, C_17:0_). Monoacylglycerols and diacylglycerols, detected in solvent extracts, were also evidence of the partial degradation of TAGs. The molecular profile extracted from the unused vessel coated with beef tallow was dominated by a moderately broad distribution of TAGs (T_46_–T_54_), typical of ruminant carcass fats [28,29], after SE. The free fatty acid profile, after SE or AE, consisted mainly of C_16:0_ and C_18:0_, with much smaller amounts of C_18:1_, and linear and branched C_15:0_ and C_17:0_. The unused vessel coated with pine resin and beef tallow had a similar profile, but with a greater relative amount of C_18:1_ compared with C_18:0_, and the presence of diterpenoids related to *Pinaceae* resin, as trimethylsilyl (TMS) or methylated derivatives: primaric and isoprimaric acids, abietic acid, dehydroabietic acid (DHA), diDHA, 7-oxo-DHA and pimara-8,15-dien-18-oic acid [29].

In short, the three coated but unused vessels absorbed a signal that enabled the coating product to be identified from the molecular data, despite being applied at high temperature and after preparatory removal of the outer surface, as had been shown for lard [2].

##### 3.2.2.2. Thermal transformation markers

Thermal transformation markers were detected in all unused coated pottery vessels. These included: ketones (K_31_, K_33_ and K_35_), produced by condensation of saturated fatty acids [30,31]; and APAAs (C_18_ and traces of C_20_) resulting from the cyclization of unsaturated fatty acids [10,32]. The identification of ketones and APAAs confirms the formation of thermal transformation markers during the coating of hot pottery surfaces with fatty products, as previously shown [2], including APAAs that have not yet been attributed to heating during the coating process. These observations are significant, as thermal transformation markers are generally used in the published literature to identify food cooking in pots [10,32,33], but could instead be formed during the use of organic products for post-firing treatments.

Additionally, a series of even and odd-numbered short-chain acids were present in unused and coated pottery vessels, but in much lower proportions than previously observed in experiments with lard [2]. This may be due to differences in the composition of the pottery fabrics or the temperatures to which the coating agents were exposed. Ketones did not appear in any of the milk-coated pots, suggesting that they are formed only if the coating agent is sufficiently concentrated in lipids (e.g. pure fats such as lard or beef tallow).

Interestingly, no transformation markers were detected in the uncoated pots that have been used to cook various fatty commodities. This may be due to the higher temperatures used for coating (300–500°C) compared with cooking temperatures less than 300°C [2,30,31]. Therefore, our results confirmed that caution should be taken when ketones and APAAs are observed in archaeological samples, as they can either reflect the technological processes or cooking processes, and both should be carefully considered in interpretation.

### 3.2.3. Coated vessels with organic content

#### 3.2.3.1. Molecular profiles

**Figure 5.**
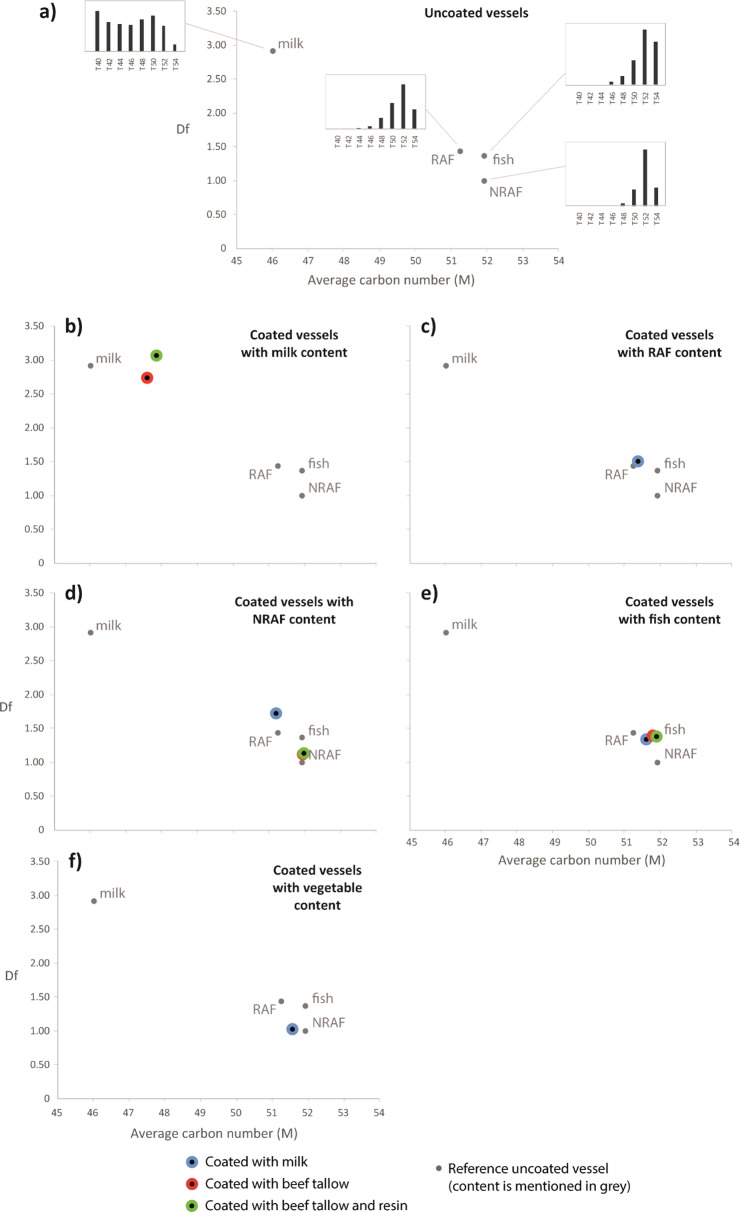
TAG distribution of samples: (a) uncoated vessels, (b–f) coated vessels, as a function of their average carbon number (M) and their dispersion factor (Df) (calculated on the range T_40_–T_54_, from [21]).

In coated vessels, some elements of the coating can still be seen, for example, diterpenoids from pine resin persisted despite the subsequent cooking of products in the vessel. The presence of diterpenoids as an indication of the use of pine resins as a coating product is often considered in archaeological ceramic studies [16]. However, in the majority of cases the molecular extracts generally originate from the contents rather than the coating agent, particularly in terms of TAG profiles, confirming the results previously obtained on lard-coated vessels [2]. All samples used to contain milk (samples 9 and 18) showed a broad TAG profile (T_40_–T_54_) characteristic of dairy products [15], despite being coated with beef tallow with or without pine resin (figure 4b and 5b). The vessel used to cook ruminant carcass fat (sample 2) showed a typical ruminant carcass fat TAG distribution from T_46_ to T_54_ and centred on T_52_ [6,12,15], despite the use of milk as a coating product (figure 5c). The profile of TAGs in the coated vessels used for cooking fish (samples 4, 11, 20) closely resembled that of the uncoated vessel (figure 5e), potentially due to challenges in identifying unsaturated TAGs. Additionally, the tallow-coated vessels used to cook non-ruminant fats (samples 10 and 19) showed a typical non-ruminant pattern (narrow distribution T_48_–T_54_, large predominance of T_52_; figure 5d) [23].

However, some samples are exceptions here. The milk-coated vessel used to cook non-ruminant fat (sample 3) showed a TAG pattern closer to that of ruminant carcass fat (figure 5d), probably resulting from a mixture of milk and non-ruminant TAG distributions. The sample that was used to contain mixed vegetable products did not yield TAG profiles when cooked without coating (sample 39) or after coating with beef tallow (sample 12). However, it did when coated with milk (sample 5), but this profile reflects NRAF rather than dairy products (figure 5f) [23]. It is possible that this sample was either contaminated during the cooking process or that the TAG distribution is made up of unsaturated TAGs which could not be properly distinguished using the conventional GC-MS techniques applied here.

#### 3.2.3.2. Single compound stable carbon isotopes

**Figure 6.**
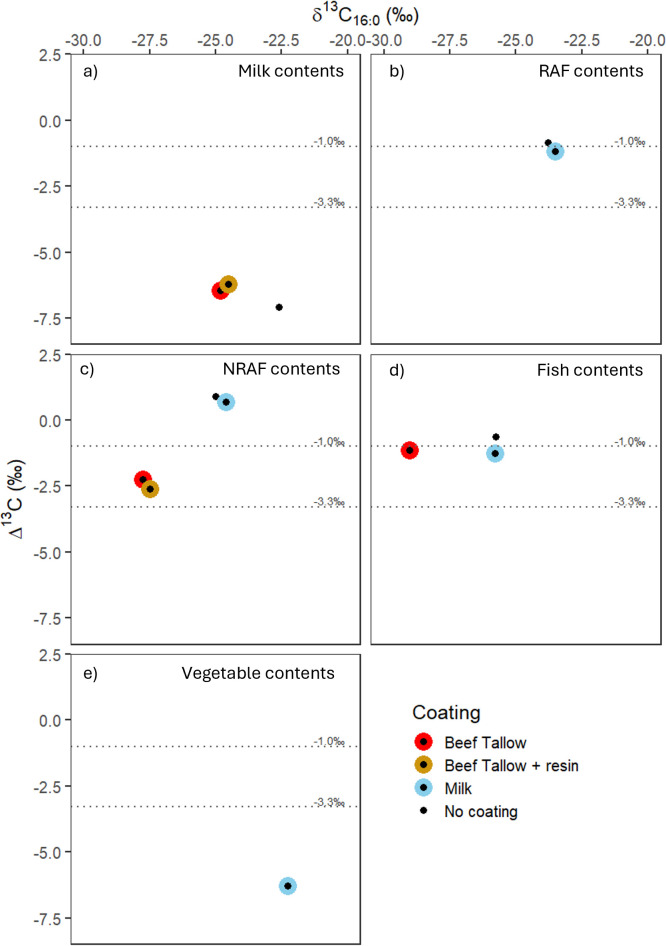
Plot of Δ^13^C against δ^13^C_16:0_ from experimental pots with different contents: (a) milk contents, (b) RAF contents, (c) NRAF contents, (d) fish contents and (e) vegetable contents; and coated with different products.

For milk-coated vessels, single compound stable carbon isotope values are always close to their content (figure 6b–d, blue dots), except when low lipid sources were cooked in the vessel. For example, the milk-coated vessel with vegetable content (sample 5) shows a Δ^13^C < -3.3‰, typical of dairy products [6,12,14] (figure 6e). In beef tallow-coated vessels and resin and beef tallow-coated vessels, the isotope values can sometimes reflect the content (e.g. milk in samples 9 and 18; figure 6a), while at other times the δ^13^C values arise from a mixture of the coating agent and the contents (e.g., fish in sample 11; figure 6d, red dot). In this case, the Δ^13^C value aligns with both fish and ruminants, but the δ^13^C value of C_16:0_ is depleted in ^13^C, indicating a contribution from terrestrial resources. Alternatively, when there was a non-ruminant content, the δ^13^C values reflected the coating agent (beef tallow in samples 10 and 19; figure 6c, red and brown dots), highlighting the complicated interaction between the coating agent and the contents of the ceramic. The influence of beef tallow coating on the δ^13^C values of these samples is probably due to the higher lipid content of this product, as demonstrated in the higher lipid yields obtained from unused pots coated with beef tallow in comparison with unused pots coated with milk (electronic supplementary material, S3).

## 4. Conclusions

This new series of experiments highlights the importance of considering the impact of post-firing coatings on our interpretation of organic residues extracted from archaeological ceramics. Here, we have further confirmed that the application of fatty products to hot pottery surfaces results in the absorption of molecular residues on the ceramic walls [2]. The experiments showed that molecular and isotopic analysis can often reliably identify the contents of pots, without interference from the surface coating (table 1). However, our analyses have sometimes shown a molecular or isotopic signal resulting from a mixture of the signals from the organic contents and the coating product. These mixtures may be due to low-fat contents (e.g. vegetables), high-fat coating agents (e.g. beef tallow), but also to local variations in the absorption of the coating product or the product contained (heterogeneity of the clay paste, varying surface temperatures during coating or use, etc.). It was only possible here to identify mixed signals through the application of a multifaceted organic residue extraction (AE and SE) and analysis method (molecular and isotope analyses).

**Table 1.** Summary of interpretations in coated and used vessels.

Sample no.	Sealing	Content	Interpretation from	
			Molecular data (TAGs)	Isotopes
2	milk	ruminant	content	content
3	milk	non-ruminant	mixed signal	content
4	milk	fish	content	content
5	milk	vegetable	other	coating
9	beef tallow	milk	content	content
10	beef tallow	non-ruminant	content	coating
11	beef tallow	fish	content	mixed signal
12	beef tallow	vegetable	no information	no information
18	resin + beef tallow	milk	content	content
19	resin + beef tallow	non-ruminant	content	coating
20	resin + beef tallow	fish	content	no information

Importantly, we have shown that during this step in the pottery *chaîne opératoire* produces thermal transformation markers (ketones and odd and even short-chain fatty acids), including APAAs, the production of which has been shown for the first time in post-firing coating experiments, after the application of an AE method. The identification of thermal transformation markers only in coated vessels and not in uncoated vessels has raised questions regarding their formation and subsequent interpretations for cooking organic products in archaeological ceramics.

We suggest that coating ceramic surfaces with fatty products might prevent the molecules of the contents from binding strongly to the pottery, which could explain the similar extraction yields between AE and SE in coated ceramics. However, this hypothesis will need to be confirmed through additional degradation experiments and further isotope measurements of both the AE and SE to better understand the origin of these lipids*.*

Therefore, to verify that the molecular and isotopic signal extracted from the archaeological ceramics corresponds to the contents of the pots, we call on the ORA community to systematically check for the presence of surface coating by employing and combining the following approaches:

(1) To carry out routine visual observation of surfaces and edges to identify evidence of coating on the surface of the ceramics [2].
(2) To employ a multifaceted ORA approach (SE and AE, molecular and isotope analyses).
(3) To routinely look for thermal transformation markers and to consider alternative explanations for their formation.
(4) To measure, report and compare the extraction yields obtained from AE and SE methods.

Future work must be carried out to investigate the impact of post-burial degradation on our identification of post-firing coatings to further understand these results and applications to the interpretation of archaeological ceramic contents. In addition, these results, which are based on only two experimental cooking events, should now be confirmed by additional experiments involving multiple cooking events and/or the analysis of ethnographic vessels to account for potential lipid turnover due to use [1]. Nonetheless, these experiments have highlighted several results that should be considered in the interpretation of archaeological ceramics.

## Data Availability

All data associated with this article is available in the supplementary materials reported here [34] and in the Dryad repository [25].

## References

[1] Miller MJ *et al*. 2020 Interpreting ancient food practices: stable isotope and molecular analyses of visible and absorbed residues from a year-long cooking experiment. Sci. Rep. **10**, 13704. (10.1038/s41598-020-70109-8) 32855436 PMC7452889

[2] Drieu L, Lepère C, Regert M. 2020 The missing step of pottery chaîne opératoire: considering post-firing treatments on ceramic vessels using macro- and microscopic observation and molecular analysis. J. Archaeol. Method Theory **27**, 302–326. (10.1007/s10816-019-09428-8)

[3] Romanus K, Baeten J, Poblome J, Accardo S, Degryse P, Jacobs P, De Vos D, Waelkens M. 2009 Wine and olive oil permeation in pitched and non-pitched ceramics: relation with results from archaeological amphorae from Sagalassos, Turkey. J. Archaeol. Sci. **36**, 900–909. (10.1016/j.jas.2008.11.024)

[4] Diallo B, Vanhaelen M, Gosselain OP. 1995 Plant constituents involved in coating practices among traditional African potters. Experientia **51**, 95–97. (10.1007/BF01964928)

[5] Turini M, Mayor A, Vieugué J, Delvoye A, Sall M, Regert M, Drieu L. In press. Unravelling ceramic content and organic coatings in Senegalese ethnographic pottery vessels. J. Archaeol. Sci. Rep.

[6] Copley MS, Berstan R, Dudd SN, Aillaud S, Mukherjee AJ, Straker V, Payne S, Evershed RP. 2005 Processing of milk products in pottery vessels through British prehistory. Antiquity **79**, 895–908. (10.1017/s0003598x00115029)

[7] Craig OE, Taylor G, Mulville J, Collins MJ, Parker Pearson M. 2005 The identification of prehistoric dairying activities in the Western Isles of Scotland: an integrated biomolecular approach. J. Archaeol. Sci. **32**, 91–103. (10.1016/j.jas.2004.06.009)

[8] Evershed RP, Heron C, Charters S, Goad LJ. 1992 The survival of food residues: new methods of analysis, interpretation and application. Proc. Br. Acad. **77**, 187–208.

[9] Correa-Ascencio M, Evershed RP. 2014 High throughput screening of organic residues in archaeological potsherds using direct acidified methanol extraction. Anal. Methods **6**, 1330. (10.1039/c3ay41678j)

[10] Hansel FA, Copley MS, Madureira LAS, Evershed RP. 2004 Thermally produced ω-(o-alkylphenyl)alkanoic acids provide evidence for the processing of marine products in archaeological pottery vessels. Tetrahedron Lett. **45**, 2999–3002. (10.1016/j.tetlet.2004.01.111)

[11] Öhlinger B, Ludwig S, Forstenpointner G, Thanheiser U. 2022 Lifting the lid: cooking pots and ritual consumption practices at Monte Iato (western Sicily, sixth–mid-fifth century BC). J. Mediterr. Archaeol. **34**, 165–192. (10.1558/jma.21979)

[12] Craig OE *et al*. 2013 Earliest evidence for the use of pottery. Nature **496**, 351–354. (10.1038/nature12109) 23575637

[13] Charters S, Evershed RP, Goad LJ, Leyden A, Blinkhorn PW, Denham V. 1993 Quantification and distribution of lipid in archaeological ceramics: implications for sampling potsherds for organic residue analysis and the classification of vessel use. Archaeometry **35**, 211–223. (10.1111/j.1475-4754.1993.tb01036.x)

[14] Craig OE, Allen RB, Thompson A, Stevens RE, Steele VJ, Heron C. 2012 Distinguishing wild ruminant lipids by gas chromatography/combustion/isotope ratio mass spectrometry. Rapid Commun. Mass Spectrom. **26**, 2359–2364. (10.1002/rcm.6349) 22956328

[15] Dudd SN, Evershed RP. 1998 Direct demonstration of milk as an element of archaeological economies. Science **282**, 1478–1481. (10.1126/science.282.5393.1478) 9822376

[16] Lundy J *et al*. 2021 New insights into early medieval Islamic cuisine: organic residue analysis of pottery from rural and urban Sicily. PLoS One **16**, e0252225. (10.1371/journal.pone.0252225) 34106970 PMC8189454

[17] Reber EA, Kerr MT, Whelton HL, Evershed RP. 2019 Lipid residues from low‐fired pottery. Archaeometry **61**, 131–144. (10.1111/arcm.12403)

[18] Dudd SN, Evershed RP, Gibson AM. 1999 Evidence for varying patterns of exploitation of animal products in different prehistoric pottery traditions based on lipids preserved in surface and absorbed residues. J. Archaeol. Sci. **26**, 1473–1482. (10.1006/jasc.1998.0434)

[19] Evershed RP. 1993 Biomolecular archaeology and lipids. World Archaeol. **25**, 74–93. (10.1080/00438243.1993.9980229) 16471029

[20] Lucquin A, Colonese AC, Farrell TFG, Craig OE. 2016 Utilising phytanic acid diastereomers for the characterisation of archaeological lipid residues in pottery samples. Tetrahedron Lett. **57**, 703–707. (10.1016/j.tetlet.2016.01.011)

[21] Mirabaud S, Rolando C, Regert M. 2007 Molecular criteria for discriminating adipose fat and milk from different species by nanoESI MS and MS/MS of their triacylglycerols: application to archaeological remains. Anal. Chem. **79**, 6182–6192. (10.1021/ac070594p) 17637040

[22] Evershed RP, Dudd SN, Copley MS, Berstan R, Stott AW, Mottram H, Buckley SA, Crossman Z. 2002 Chemistry of archaeological animal fats. Acc. Chem. Res. **35**, 660–668. (10.1021/ar000200f) 12186571

[23] Mukherjee AJ, Berstan R, Copley MS, Gibson AM, Evershed RP. 2007 Compound-specific stable carbon isotopic detection of pig product processing in British Late Neolithic pottery. Antiquity **81**, 743–754. (10.1017/s0003598x00095703)

[24] Roffet-Salque M, Lee MRF, Timpson A, Evershed RP. 2017 Impact of modern cattle feeding practices on milk fatty acid stable carbon isotope compositions emphasise the need for caution in selecting reference animal tissues and products for archaeological investigations. Archaeol. Anthropol. Sci. **9**, 1343–1348. (10.1007/s12520-016-0357-5)

[25] Lundy J, Haas J, Öhlinger B, Drieu L. 2025 Beyond the surface: untangling molecular complexity in organic residue analysis of coated archaeological ceramics. Dryad Digital Repository (10.5061/dryad.51c59zwmc) PMC12520769 41098819

[26] Charters S, Evershed RP, Quye A, Blinkhorn PW, Reeves V. 1997 Simulation experiments for determining the use of ancient pottery vessels: the behaviour of epicuticular leaf wax during boiling of a leafy vegetable. J. Archaeol. Sci. **24**, 1–7. (10.1006/jasc.1995.0091)

[27] Drieu L, Regert M, Mazuy A, Vieugué J, Bocoum H, Mayor A. 2022 Relationships between lipid profiles and use of ethnographic pottery: an exploratory study. J. Archaeol. Method Theory **29**, 1294–1322. (10.1007/s10816-021-09547-1)

[28] Colombini MP, Modugno F, Ribechini E. 2005 Direct exposure electron ionization mass spectrometry and gas chromatography/mass spectrometry techniques to study organic coatings on archaeological amphorae. J. Mass Spectrom. **40**, 675–687. (10.1002/jms.841) 15739159

[29] Pollard AM, Heron C. 2008 The chemistry and use of resinous substances. In Archaeological chemistry (eds AM Pollard, C Heron, RD Gillard), pp. 235–269. Cambridge, UK: The Royal Society of Chemistry.

[30] Raven AM, van Bergen PF, Stott AW, Dudd SN, Evershed RP. 1997 Formation of long-chain ketones in archaeological pottery vessels by pyrolysis of acyl lipids. J. Anal. Appl. Pyrolysis **40–41**, 267–285. (10.1016/S0165-2370(97)00036-3)

[31] Evershed RP, Stott AW, Raven A, Dudd SN, Charters S, Leyden A. 1995 Formation of long-chain ketones in ancient pottery vessels by pyrolysis of acyl lipids. Tetrahedron Lett. **36**, 8875–8878. (10.1016/0040-4039(95)01844-8)

[32] Bondetti M, Scott E, Courel B, Lucquin A, Shoda S, Lundy J, Labra‐Odde C, Drieu L, Craig OE. 2021 Investigating the formation and diagnostic value of ω ‐(o ‐alkylphenyl)alkanoic acids in ancient pottery. Archaeometry **63**, 594–608. (10.1111/arcm.12631) 34219747 PMC8247306

[33] Shoda S, Lucquin A, Sou CI, Nishida Y, Sun G, Kitano H, Son J ho, Nakamura S, Craig OE. 2018 Molecular and isotopic evidence for the processing of starchy plants in Early Neolithic pottery from China. Sci. Rep. **8**, 17044. (10.1038/s41598-018-35227-4) 30451924 PMC6242940

[34] Lundy J, Haas J, Öhlinger B, Drieu L. 2025 Supplementary material from: Beyond the Surface: Untangling Molecular Complexity in Organic Residue Analysis of Coated Archaeological Ceramics. Figshare. (10.6084/m9.figshare.c.8026211) PMC12520769 41098819

